# A Pseudoenzymatic Regulatory Role of the Noncatalytic Subunit of the Neurotoxin Vipoxin at Arachidonic-Acid-Containing Membrane Interfaces

**DOI:** 10.3390/membranes16070241

**Published:** 2026-07-16

**Authors:** Svetla Petrova, Kristina Mircheva, Evgenia Sotirovska, Nikolay Alexandrov Grozev, Kirilka Stefanova Mladenova, Pavel Videv, Jordan Doumanov, Konstantin Balashev

**Affiliations:** 1 Laboratory of Enzymology, Department of Biochemistry, Faculty of Biology, Sofia University “St. Kl. Ohridski”, 1164 Sofia, Bulgaria; spetrova@biofac.uni-sofia.bg (S.P.); essotirovs@uni-sofia.bg (E.S.); k_mladenova@biofac.uni-sofia.bg (K.S.M.); pvidev@biofac.uni-sofia.bg (P.V.); doumanov@biofac.uni-sofia.bg (J.D.); 2 Laboratory of Biophysical Chemistry, Department of Physical Chemistry, Faculty of Chemistry and Pharmacy, Sofia University “St. Kl. Ohridski”, 1164 Sofia, Bulgaria; fhkm@chem.uni-sofia.bg (K.M.); fhng@chem.uni-sofia.bg (N.A.G.)

**Keywords:** Vipoxin, phospholipase A_2_ (PLA_2_), pseudoenzyme, Langmuir monolayer, membrane packing, lipid–protein interactions, snake venom neurotoxins

## Abstract

Secreted phospholipases A_2_ (sPLA_2_s) act at lipid interfaces where enzymatic turnover is strongly influenced by membrane packing and interfacial physicochemical conditions. Vipoxin, a heterodimeric neurotoxin from *Vipera ammodytes meridionalis*, is one such complex, comprising a catalytically active sPLA_2_ subunit (VBC) and a catalytically impaired homolog, VAC, suggesting a pseudoenzymatic regulatory role. Using SAPC Langmuir monolayers as a model of arachidonic-acid-containing membranes, we monitored the compensated monolayer area change, ΔA(t), under barostatic conditions as an integrated readout of the interfacial behavior of Vipoxin and its isolated subunits. The responses revealed pronounced modulation by surface pressure and by the acidic acetate versus basic Tris-HCl subphase environment: VBC retained high catalytic competence under both conditions, whereas Vipoxin displayed greater environmental sensitivity, consistent with VAC-dependent modulation of enzyme–membrane coupling. VAC, although lacking canonical catalytic activity, produced measurable interfacial effects under acidic conditions and high lateral pressure. Analysis using the interfacial quality parameter $Q_{m}$ demonstrated that VAC modifies the pressure dependence of the heterodimer and stabilizes interfacial accommodation of VBC. These findings indicate that VAC functions as a pseudoenzymatic regulatory subunit whose role emerges from dynamic coupling between enzymatic activity and lipid interfacial organization.

## 1. Introduction

Biological membranes are dynamic, heterogeneous assemblies whose structural and functional properties are continuously reshaped by lipid composition, enzymatic activity, and local physicochemical conditions. Among the enzymes that actively remodel membrane lipids, secreted phospholipases A_2_ (sPLA_2_s) play a leading role, catalyzing the hydrolysis of glycerophospholipids to yield lysophospholipids and free fatty acids [1,2]. These reaction products are themselves highly surface-active and biologically potent, capable of inducing profound changes in membrane organization, interfacial packing, and lateral heterogeneity [3,4]. Consequently, sPLA_2_ activity is intrinsically governed by the evolving physical state of the membrane interface on which it acts. Because these enzymes function at structurally dynamic lipid interfaces, they provide particularly valuable systems for investigating the interplay between membrane organization, interfacial activation, and lipid-protein interactions.

Snake venoms provide a rich source of structurally diverse sPLA_2_s and sPLA_2_-related proteins and have long served as model systems for investigating lipid-protein interactions at membrane interfaces [5]. One particularly informative system is Vipoxin, a heterodimeric neurotoxin isolated from the venom of the Bulgarian nose-horned viper *Vipera ammodytes meridionalis* [6,7,8]. Vipoxin consists of a catalytically active Ca^2+^-dependent sPLA_2_ subunit (VBC) and a catalytically impaired homolog, VAC, associated through non-covalent interactions. Although the two subunits share substantial sequence and structural homology, VAC lacks the conserved catalytic His48 residue, which is replaced by Gln48 [9,10,11,12,13,14]. Moreover, crystal structures of Vipoxin and isolated monomeric VAC showed that the subunits associate through complementary hydrophobic interfaces reinforced by electrostatic interactions, forming a stable heterodimer in which VAC partially shields the catalytic site of VBC [12,15,16]. Consequently, VAC was initially described as an inhibitory subunit because the PLA_2_ activity of heterodimeric Vipoxin was lower than that of isolated VBC [7,17]. However, subsequent studies demonstrated that this interpretation is incomplete. In addition to contributing to the structural stability of the complex, VAC modulates the membrane-binding specificity of VBC, shifting its preference from predominantly presynaptic sites to postsynaptic membrane regions [11,14]. These findings support the view that VAC functions as a pseudoenzymatic regulatory component whose biological role emerges primarily through membrane-mediated interactions rather than direct catalysis, consistent with the broader concept of catalytically impaired PLA_2_ homologs acting as functional regulators at biological interfaces [18,19]. Nevertheless, several catalytically inactive PLA_2_ homologs retain significant neurotoxic, myotoxic, or anticoagulant effects, indicating that non-enzymatic mechanisms—such as specific membrane interactions or distinct pharmacological sites—play a critical role in their biological function [20,21,22].

An important yet insufficiently explored aspect of Vipoxin regulation concerns the chemical nature of the phospholipid substrate and the physicochemical properties of its hydrolysis products. Arachidonic acid (AA)-containing phospholipids, such as 1-stearoyl-2-arachidonoyl-*sn*-glycero-3-phosphocholine (SAPC), are critical components of cellular membranes. They regulate membrane fluidity and serve as essential precursors for eicosanoid signaling networks and inflammatory responses [3,23]. While the release of AA in mammals is primarily attributed to intracellular cytosolic phospholipases (e.g., GIVA cPLA_2_), secreted snake venom PLA_2_s also exhibit potent activity against these substrates. For instance, ammodytoxin—a monomeric PLA_2_ isolated from the venom of the closely related *Vipera ammodytes ammodytes*—efficiently catalyzes AA release to induce apoptosis in motoneuronal cell lines [24]. Following enzymatic cleavage, SAPC hydrolysis releases free arachidonic acid and lysophosphatidylcholine—two amphiphilic molecules known to strongly perturb membrane packing, promote lateral heterogeneity, and exhibit pronounced pH-dependent behavior at lipid interfaces [3,23]. As a result, SAPC monolayers represent a dynamically evolving interfacial environment in which enzyme activity, membrane organization, and local physicochemical conditions are tightly coupled.

To systematically examine these interactions, the present study employs Langmuir phospholipid monolayers as a controlled interfacial model for investigating how arachidonic-acid-containing membranes modulate the interfacial activity of Vipoxin and its individual subunits [4]. For clarity, heterodimeric Vipoxin and the isolated VBC and VAC subunits are hereafter collectively referred to as Vipoxin protein forms when their common interfacial behavior is discussed; distinctions between catalytically active and catalytically impaired components are specified where mechanistically relevant. Under barostatic conditions, changes in monolayer area, $\Delta A\left(t\right)$, induced by these protein forms provide a macroscopic readout of the integrated interfacial response, including adsorption, penetration, substrate binding, catalytic turnover (where applicable), product accumulation or removal, and lipid reorganization. Accordingly, $\Delta A\left(t\right)$ is not treated as a direct product-specific measure of hydrolysis alone, but as an experimentally accessible kinetic response of the protein–monolayer system. Particular emphasis is placed on the experimentally derived interfacial quality parameter $Q_{m}\left(\exp\right)$, extracted from the initial $\Delta A\left(t\right)$ slopes, as a normalized comparative descriptor of effective interfacial activity under conditions of pronounced monolayer heterogeneity. This framework allows us to compare how lipid substrate properties, subphase-condition-dependent interfacial behavior, and VAC-mediated pseudoenzymatic modulation shape phospholipase action at biologically relevant membrane interfaces.

## 2. Materials and Methods

### 2.1. Materials

1-Stearoyl-2-arachidonoyl-sn-glycero-3-phosphocholine (SAPC; purity ≥ 99%, $M_{w}=810.1\;g{mol}^{-1}$) 1,2-dipalmitoyl-sn-glycero-3-phosphocholine (DPPC; purity ≥ 99%, $M_{w}=734.05\;g{mol}^{-1}$) were purchased from Sigma-Aldrich (St. Louis, MO, USA) and used as phospholipid substrates. Chloroform (Merck, Darmstadt, Germany) was used as the spreading solvent for monolayer preparation.

Two aqueous buffer systems were employed:

(i) Basic buffer (pH 8.0): 30 mM Tris-HCl, 150 mM NaCl, 10 mM ${CaCl}_{2}$
(ii) Acidic buffer (pH 5.5): 50 mM sodium acetate, 150 mM NaCl, 10 mM ${CaCl}_{2}$

The two buffer systems were selected to provide stable acidic and basic subphase conditions compatible with phospholipase A_2_ activity and monolayer measurements. Both buffers contained identical concentrations of NaCl and CaCl_2_ in order to maintain comparable background electrolyte composition and calcium availability. However, because the buffering species differed between the two conditions, the observed differences between pH 5.5 and pH 8.0 cannot be attributed exclusively to proton activity and may also include buffer-specific contributions such as ion association, hydration effects, or electrostatic screening. Therefore, comparisons between pH 5.5 and pH 8.0 are interpreted throughout the manuscript as differences between acidic acetate and basic Tris-HCl subphase conditions, with pH considered the principal but not exclusive variable.

All buffer components were obtained from Merck (Merck, Darmstadt, Germany. $\beta -Cyclodextrin\;(\beta -CD;\;M_{w}=1135\;g\;mol^{-1}$; Sigma-Aldrich) was used without further purification and was added to the aqueous subphase at a final concentration of $1\;mg{mL}^{-1}$ in all barostatic interfacial kinetic experiments. β−CD was included to facilitate the extraction of poorly soluble long-chain hydrolysis products, where generated, from the monolayer and to minimize their accumulation at the interface.

Double-distilled water was used for the preparation of all solutions and for the cleaning of the Langmuir–Blodgett trough.

### 2.2. Isolation and Purification of Vipoxin and Its Components

Vipoxin was isolated from crude venom of *Vipera ammodytes meridionalis* using an established multi-step chromatographic protocol described previously [9,10]. Briefly, crude venom was fractionated by cation-exchange chromatography on SP-Sephadex C-50. The heterodimeric Vipoxin complex was then dissociated, and its individual subunits were separated by fast protein liquid chromatography (FPLC) on a Mono S cation-exchange column equilibrated in 0.1 M acetate buffer (pH 4.5) containing 6 M urea. Because heterogeneity may occur within the acidic VAC-containing fractions, these fractions were further purified by anion-exchange chromatography on a Mono Q column in order to minimize possible carryover of catalytically active material. The purified Q4 VAC fraction, previously characterized by UFLC and SDS-PAGE, was used for the VAC experiments. Protein purity was assessed by SDS-PAGE, and protein concentration was determined before use. In addition, PLA_2_ activity checks were performed before the interfacial experiments to verify the functional integrity of the catalytically active preparations. The final concentration used in all interfacial kinetic experiments was 6 nM for heterodimeric Vipoxin, VBC, and VAC.

### 2.3. Interfacial Measurements at the Air-Water Interface

#### 2.3.1. Langmuir Trough Setup

Interfacial phospholipase $A_{2}$ activity was investigated using a Langmuir barostatic balance (KSV NIMA 2000, Biolin Scientific, Espoo, Finland). Surface pressure (π) was monitored by the Wilhelmy plate method using a platinum plate. All experiments were conducted at 24±0.5 °C. Each experiment was repeated at least three times to ensure reproducibility.

#### 2.3.2. Monolayer Preparation and Compression

SAPC monolayers were spread from a chloroform solution (${1\;mgmL}^{-1}$) onto the air–water interface. After 10 min to allow evaporation of the organic solvent, the monolayer was compressed at a constant barrier speed of $10\;{cm}^{2}{min}^{-1}$ to the desired surface pressure. Upon reaching the target surface pressure, the system was switched to barostatic mode, and the SAPC monolayer was equilibrated for 30–45 min prior to injection of the respective Vipoxin protein form. This extended equilibration period allowed the highly unsaturated SAPC monolayer to reach a stable barostatic baseline before kinetic measurements were initiated. During this period, monolayer stability was monitored from the constancy of surface pressure and the absence of measurable compensated area drift beyond experimental error.

#### 2.3.3. Experimental Setup

**Figure 1:**
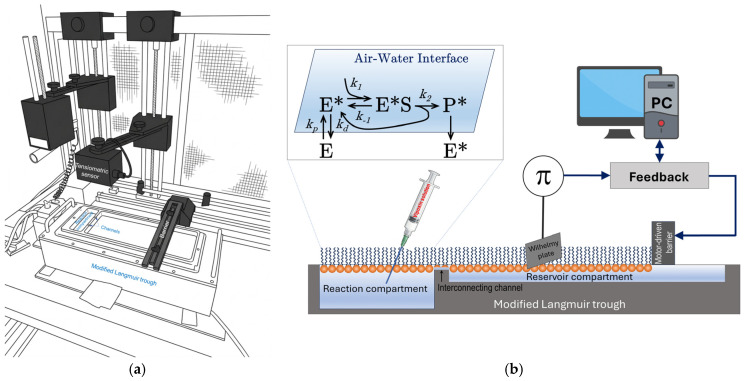
Experimental setup and conceptual framework for interfacial enzymatic measurements. (**a**) Schematic illustration of the experimental Langmuir setup used for interfacial phospholipase measurements. The system utilizes a Langmuir trough (KSV NIMA 2000 (Biolin Scientific, Espoo, Finland)) equipped with one movable barrier for monolayer compression and a Wilhelmy plate connected to a high-sensitivity tensiometric sensor for continuous surface pressure (π) monitoring. The trough is adapted to include separated compartments and controlled injection pathways, allowing precise manipulation of the lipid monolayer and subphase conditions under strict barostatic control. (**b**) Conceptual scheme of the experimental configuration and simplified interfacial kinetic model. SAPC monolayers are formed at the air–water interface of a modified Langmuir trough comprising a reaction compartment and a reservoir compartment, hydraulically connected through an interconnecting channel that ensures continuity of the lipid monolayer and pressure equilibration between compartments. Surface pressure is maintained under barostatic conditions via a feedback-controlled motor-driven barrier system monitored by a Wilhelmy plate. The enzyme solution (Vipoxin or its isolated subunits) is injected into the aqueous subphase beneath the monolayer in the reaction compartment. The enzyme partitions from the aqueous phase (E) to the interface ($E^{*}$), forming an interfacially activated state. Subsequent binding to the lipid substrate (S) yields the enzyme–substrate complex (ES), followed by catalytic turnover ($k_{2}$) to generate interfacial products ($P^{*}$). The adsorption–desorption equilibrium is described by the rate constants $k_{p}$ and $k_{d}$, while $k_{1}$ and $k_{-1}$ represent two-dimensional interfacial binding dynamics. The barostatic response, recorded as the compensated change in monolayer area $\Delta A\left(t\right)$, reflects the net macroscopic balance among lipid hydrolysis, product desorption, enzyme adsorption, and interfacial reorganization.

Interfacial phospholipase A_2_ activity was investigated using a Langmuir barostatic balance (KSV NIMA 2000, Biolin Scientific, Espoo, Finland), as schematically illustrated in Figure 1.

All experiments were conducted at 24 ± 0.5 °C, and each measurement was repeated at least three times to ensure reproducibility.

#### 2.3.4. Interfacial Kinetic Experiments with Vipoxin Protein Forms

Interfacial kinetic experiments with SAPC monolayers were initiated by injecting heterodimeric Vipoxin, VBC, or VAC into the aqueous subphase beneath the lipid monolayer under strictly controlled barostatic conditions (Figure 1b). Consistent with the terminology introduced above, these three species are collectively referred to as Vipoxin protein forms when their common interfacial behavior is discussed.

Interfacial kinetics were followed by recording the compensated change in monolayer area, ΔA(t), as a function of time following injection t = 0. Measurements were performed at surface pressures of $\pi \;=\;5,\;10,\;15,\;and\;20\;mN\;m^{-1}$ using either pH 8.0 or pH 5.5 buffer as the subphase.

Under constant surface-pressure conditions, perturbations induced by the respective Vipoxin protein form are continuously compensated by barrier movement. The resulting ΔA(t) signal represents an integrated macroscopic response of the Vipoxin protein form–monolayer system rather than a direct measure of phospholipid hydrolysis alone. Positive ΔA(t) values are consistent with the net removal of surface-active material from the interface, whereas negative ΔA(t) values indicate dominance of interfacial incorporation processes, such as adsorption and/or penetration into the lipid phase. Thus, ΔA(t) reports the balance among adsorption, penetration, lipid rearrangement, catalytic turnover, where applicable, and product removal under barostatic conditions.

### 2.4. Kinetic Analysis and Interfacial Enzymology Framework

The interfacial response of the Vipoxin protein form–monolayer system was quantified from the experimental ΔA(t) traces recorded under barostatic conditions. Interfacial activity at the lipid interface was evaluated using the interfacial quality parameter $Q_{m}$, originally introduced by Verger within the framework of interfacial enzymology [25]. While the basic sequence of interfacial catalysis can be schematically summarized by the simplified partitioning and turnover model shown in Figure 1b, the action of phospholipases on complex lipid monolayers often requires a more comprehensive theoretical treatment.

**Figure 2:**
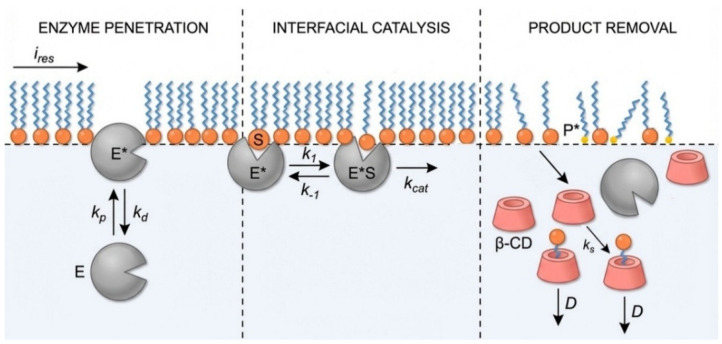
Schematic representation of the interfacial kinetic mechanism adapted for phospholipase A_2_ activity on SAPC monolayers in the presence of β-cyclodextrin. The water-soluble enzyme (E) reversibly partitions into the lipid–water interface to form an interfacially activated state ($E^{*}$), which binds substrate molecules (S) within the two-dimensional lipid phase to generate the enzyme–substrate complex ($E^{*}S$). Catalytic turnover ($k_{cat}$) yields interfacial products (P∗). Due to the poor aqueous solubility of long-chain hydrolysis products, their removal from the interface may be facilitated by complex formation with β-cyclodextrin (β-CD), followed by diffusion into the aqueous subphase. In the present study, $Q_{m}$(exp) was derived directly from the initial slopes of the ΔA(t) traces without explicit fitting of solubilization rate constants. Yellow circles denote the carboxyl groups of released free fatty acid products (predominantly arachidonic acid), whereas lysophosphatidylcholine retains the original phosphocholine headgroup.

The present analysis is therefore interpreted within the extended interfacial Michaelis–Menten formalism developed by Verger and de Haas for phospholipase action at lipid interfaces [25], and subsequently expanded by Verger, Panaiotov, and co-workers for long-chain phospholipid monolayers, where the poor aqueous solubility of reaction products necessitates explicit consideration of interfacial product removal mechanisms [26,27] (Figure 2). Within this framework, the water-soluble enzyme (E) reversibly partitions to the lipid–water interface to form an interfacially activated state ($E^{*}$), followed by two-dimensional binding to substrate molecules (S) to generate the enzyme–substrate complex ($E^{*}S$), and subsequent catalytic turnover.

For long-chain phospholipids such as SAPC, the poor aqueous solubility of hydrolysis products introduces additional complexity, as these products tend to accumulate at the interface and alter the apparent kinetic response. As illustrated in Figure 2, their removal may involve auxiliary processes such as complexation with β-cyclodextrin (β-CD) followed by diffusion into the aqueous subphase. However, quantitative treatment of these processes requires assumptions regarding interfacial molecular areas and solubilization rate constants, which are difficult to define reliably for heterogeneous monolayer systems.

For clarity, the experimentally derived parameter used throughout this study is denoted $Q_{m}\left(\exp\right)$, whereas $Q_{m}\left(\mathrm{formal}\right)$ refers to values estimated using the complete Verger–Panaiotov kinetic formalism. Because rigorous mechanistic treatment of heterogeneous SAPC monolayers requires assumptions regarding interfacial molecular areas, product accumulation, and β-cyclodextrin-mediated solubilization kinetics, the principal comparative descriptor employed in the present work was the experimentally derived parameter $Q_{m}\left(\exp\right)$. $Q_{m}\left(\exp\right)$ was calculated directly from the initial slopes of the experimental ΔA traces according to

(1) $$Q_{m}\left(\exp\right)=\frac{1}{A_{lip},C_{E}}{\left(\frac{d\Delta A}{dt}\right)}_{0},$$

where $A_{lip}$ is the mean molecular area of SAPC at the corresponding surface pressure; $C_{E}$ is the enzyme concentration in the aqueous subphase; and ${\left(\frac{d\Delta A}{dt}\right)}_{0}$ is the initial rate of barostatic area compensation. Under this normalization, the compensated macroscopic area change is converted into an effective rate of lipid turnover per enzyme concentration, yielding units consistent with the interfacial quality formalism.

## 3. Results

### 3.1. Interfacial Phase Behavior and Mechanical Properties of SAPC Monolayers

To relate enzymatic activity to monolayer structure, the phase behavior of SAPC was characterized using surface pressure–area (π−A) compression isotherms at the air–water interface. To highlight the effect of acyl chain composition, SAPC was compared with the saturated phospholipid 1,2-dipalmitoyl-sn-glycero-3-phosphocholine (DPPC), a well-established reference monolayer system.

**Figure 3:**
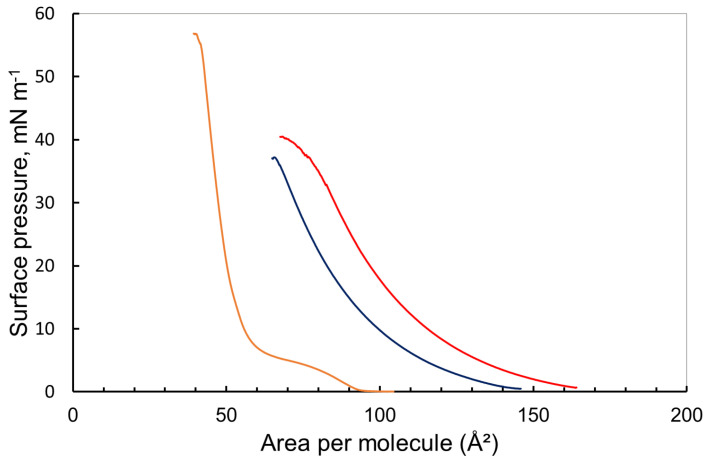
Surface pressure–area (π−A) isotherms of phospholipid monolayers at the air–water interface. The isotherm of the saturated phospholipid DPPC (orange) is compared with SAPC monolayers recorded on the basic Tris-HCl subphase, pH 8.0 (blue), and the acidic acetate subphase, pH 5.5 (red). DPPC exhibits a characteristic liquid-expanded/liquid-condensed (LE–LC) phase transition and forms a densely packed monolayer at low molecular areas. In contrast, SAPC displays expanded isotherms without a distinct phase-transition plateau, indicating a persistent liquid-expanded state due to the presence of the polyunsaturated arachidonoyl chain. The shift toward larger molecular areas under acidic acetate conditions indicates additional expansion of the SAPC monolayer.

As shown in Figure 3, the DPPC isotherm exhibits the characteristic features of a saturated phosphatidylcholine monolayer, including a pronounced plateau corresponding to liquid-expanded/liquid-condensed (LE–LC) phase coexistence, followed by transition into a densely packed, ordered state at low molecular areas [28,29]. In contrast, SAPC displays a distinctly different compression behavior [30]. The presence of the polyunsaturated arachidonoyl chain (20:4) prevents efficient chain packing, resulting in isotherms that are shifted to significantly larger molecular areas and lack a distinct LE–LC transition plateau. Instead, SAPC remains in a fluid, liquid-expanded (LE) state over the entire compression range investigated. Notably, the observed SAPC isotherms are in good agreement with previously reported data for arachidonic-acid-containing phosphatidylcholines (often denoted as PAPC in the literature), which similarly exhibit expanded isotherms and absence of clear phase transitions due to the high degree of chain unsaturation [30].

A comparison of SAPC isotherms recorded on the basic Tris-HCl subphase (pH 8.0) and the acidic acetate subphase (pH 5.5) reveals a systematic shift toward larger molecular areas under acidic conditions, indicating further expansion of the monolayer. Given the zwitterionic nature of the phosphocholine headgroup, this effect is unlikely to arise from direct protonation of the lipid headgroup alone. Instead, it is more plausibly associated with changes in interfacial hydration, ion association, electrostatic screening, and lateral organization. Importantly, pH-associated modifications of lipid organization are not restricted to systems undergoing direct charge neutralization. For example, studies on anionic bis(monoacylglycero)phosphate (BMP) membranes have shown that even partial protonation can induce substantial changes in hydration, headgroup orientation, and membrane morphology, leading to pronounced structural heterogeneity at acidic pH [31]. Although the molecular mechanisms differ due to the distinct chemical nature of the lipid headgroups, these findings highlight a general principle: relatively small changes in the interfacial environment can produce amplified collective effects on membrane organization and packing. In contrast to BMP membranes, where acidic pH directly modifies headgroup charge and orientation, SAPC remains largely zwitterionic over the investigated pH range. Therefore, the observed expansion of SAPC is interpreted as a subphase-condition-dependent effect, primarily associated with acidic pH but potentially influenced by buffer-specific contributions arising from the acetate versus Tris-HCl environment. The presence of a highly polyunsaturated arachidonoyl chain imposes a dominant steric and entropic constraint, favoring lateral expansion and stabilizing a persistent liquid-expanded state rather than condensation under compression.

To quantify the mechanical properties of the monolayers at biologically relevant packing densities, the isothermal area compressibility modulus ($C_{s}^{-1}$) was evaluated in the $\pi =\;20-30\;mN\;m^{-1}$ range, which approximates the lateral pressure in biological membranes:

(2) $$C_{s}^{-1}=-A\left(\frac{\partial \pi }{\partial A}\right),$$

The extracted parameters (Table 1) reveal pronounced differences in interfacial organization despite comparable magnitudes of $C_{s}^{-1}$.

Although the apparent compressibility modulus of SAPC at pH 5.5 ($\sim 100\;mN\;m^{-1}$) approaches that of condensed DPPC ($\sim 105\;mN\;m^{-1}$), the physical origins of this response are fundamentally different. DPPC reaches high surface pressures at relatively small molecular areas, and its high modulus reflects the resistance of a tightly packed, ordered monolayer to further compression. In contrast, SAPC remains in a liquid-expanded state even at elevated surface pressures. In this regime, resistance to compression arises primarily from steric and conformational constraints associated with the disordered, highly flexible arachidonoyl chains within an expanded interfacial film. Thus, similar values of $C_{s}^{-1}$ do not imply equivalent structural states but rather reflect different mechanisms of accommodating lateral stress. Importantly, the SAPC monolayer retains a high degree of lateral heterogeneity and dynamic fluctuations across the studied pressure range. This distinction is critical for interpreting enzymatic activity: unlike condensed monolayers, the expanded and disordered SAPC interface is expected to support transient packing defects and local rearrangements.

Such a fluctuation-rich interfacial environment is consistent with enhanced accessibility of the lipid substrate to interfacial enzymes. In this context, the SAPC monolayer provides a dynamic platform in which adsorption, penetration, and interfacial activation of Vipoxin protein forms can be modulated by lipid packing and by the acidic acetate versus basic Tris-HCl subphase environment, in agreement with the pressure- and subphase-condition-dependent behavior observed experimentally.

### 3.2. Effect of Monolayer Surface Pressure on Interfacial Activity

The influence of monolayer packing density on the interfacial activity of Vipoxin and its isolated subunits was investigated over a range of surface pressures at pH 5.5 and 8.0. In all experiments, the protein concentration was maintained at 6 nM, and the corresponding ΔA(t) traces were recorded following enzyme injection. Because ΔA(t) reflects the net interfacial response arising from both lipid hydrolysis and protein adsorption, the absence of positive signals at low surface pressures does not necessarily indicate loss of catalytic activity but may instead reflect dominance of adsorption-driven interfacial expansion.

**Figure 4:**
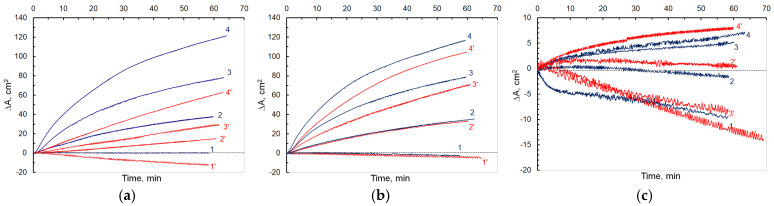
Effect of monolayer surface pressure on Vipoxin interfacial activity. Time dependence of the compensated monolayer area change, ΔA(t), recorded during interaction of SAPC monolayers with Vipoxin and its isolated subunits under strictly barostatic conditions following protein injection into the subphase (t = 0): (**a**) Heterodimeric Vipoxin; (**b**) Catalytic subunit VBC (sPLA_2_); (**c**) Non-catalytic subunit VAC. Experiments were performed at a fixed protein concentration of 6 nM and at four constant surface pressures: $1\;(\pi \;=\;5\;mN\;m^{-1});\;2\;(\pi \;=\;10\;mN\;m^{-1});\;3\;(\pi \;=\;15\;mN\;m^{-1});\;4\;(\pi \;=\;20\;mN\;m^{-1})$. Measurements at pH 8.0 (Tris-HCl buffer) are shown as solid curves (plain numbers), whereas measurements at pH 5.5 (acetate buffer) are indicated by primed numbers. Under barostatic control, positive ΔA(t) values are consistent with net removal of surface-active material from the interface, whereas negative ($\Delta A\left(t\right)$) values indicate dominance of adsorption and/or interfacial incorporation of the respective Vipoxin protein form.

For heterodimeric Vipoxin (Figure 4a), the response at the lowest surface pressure ($\pi \;=\;5\;mN\;m^{-1}$) differs qualitatively from that observed at higher packing densities. At pH 8.0, the ΔA(t) trace remains essentially constant (curve 1), indicating the absence of detectable net hydrolysis or product removal. In contrast, at pH 5.5, the corresponding trace (curve 1′) becomes negative, reflecting an apparent increase in monolayer area under barostatic control. Negative ΔA(t) values are most consistently interpreted as arising from adsorption and/or partial insertion of surface-active protein molecules, which increases surface pressure and necessitates barrier expansion to maintain constant π. At higher surface pressures ($\pi \;\geq \;10\;mN\;m^{-1}$), Vipoxin generates positive ΔA(t) traces whose slopes rise with the increase in surface pressure, indicating a transition from adsorption-dominated interfacial perturbation to productive catalytic turnover coupled to net removal of hydrolysis products. Distinct kinetic profiles are observed at pH 5.5 and pH 8.0 across the pressure range, demonstrating that subphase pH modulates the coupling between enzymatic turnover and monolayer organization.

Representative ΔA(t) traces for the catalytic subunit VBC are shown in Figure 4b. At $\pi \;=\;5\;mN\;m^{-1}$ (curves 1 and 1′), the responses at both pH values are indistinguishable within experimental error and evolve into the negative ΔA(t) region, consistent with adsorption- and/or insertion-dominated interfacial behavior. A similar overlap is observed at $\pi =10\;mN\;m^{-1}$ (curves 2 and 2′), indicating that within the expanded-to-moderately packed regime, the macroscopic interfacial response of VBC exhibits only weak pH dependence. With increasing surface pressure ($\pi \;=\;15\;and\;20\;mN\;m^{-1}$; curves 3/3′ and 4/4′), ΔA(t) becomes strongly positive and its slope increases, reflecting dominance of productive catalytic turnover and net removal of reaction products. Across this higher-pressure regime, VBC consistently exhibits larger $\Delta A\left(t\right)$ slopes in the basic Tris-HCl subphase (pH 8.0) than in the acidic acetate subphase (pH 5.5), although the magnitude of this subphase-condition dependence is substantially smaller than that observed for the Vipoxin heterodimer. This comparatively modest sensitivity to the subphase environment suggests that the isolated catalytic subunit is governed primarily by monolayer packing, whereas assembly into the heterodimer amplifies modulation by acidic versus basic subphase conditions.

VAC subunit displays a qualitatively distinct interfacial response (Figure 4c). At low surface pressures ($\pi =5-10\;mN\;m^{-1}$), ΔA(t) remains negative at both pH values, consistent with adsorption-induced monolayer expansion. No evidence of hydrolysis-driven area compensation is observed in this regime. At $\pi =15\;mN\;m^{-1}$, VAC produces minimal net area change, whereas at $\pi =20\;mN\;m^{-1}$ a small but reproducible positive ΔA(t) response is detected. Because ΔA(t) reflects the net mechanical compensation required to maintain constant surface pressure, the measured signal cannot discriminate between adsorption of VAC at the air/water interface, partial insertion into the lipid phase, or lipid displacement/rearrangement processes (i.e., competition with phospholipid molecules for interfacial area). The most conservative interpretation is therefore that VAC exhibits a weak, pressure-dependent interfacial interaction that becomes detectable only at elevated lateral pressure. This response is slightly more pronounced at pH 5.5, suggesting that acidic conditions enhance VAC’s membrane-interacting or packing-disruptive tendencies. Because VAC lacks catalytic competence, the observed ΔA(t) variations are attributed primarily to interfacial restructuring, lipid displacement, and adsorption phenomena rather than enzymatic turnover. To rationalize the origin of the negative ΔA(t) responses observed at low surface pressures, consideration of the molecular dimensions of Vipoxin and its isolated subunits provides a useful physical context. Based on crystallographic data, Vipoxin heterodimer exhibits approximate dimensions of 30 × 55 × 32 Å, whereas the isolated subunits measure roughly 12 × 40 × 25 Å [12]. Depending on interfacial orientation, these dimensions correspond to a theoretical cross-sectional footprint on the order of several hundred to over one thousand Å^2^ per molecule. Based on the SAPC compression isotherms, the molecular area of SAPC within the investigated pressure range remains substantially larger than that of saturated phosphatidylcholine monolayers and varies strongly with both surface pressure and subphase pH. At experimentally relevant surface pressures, SAPC occupies an expanded interfacial area, approximately in the range of $80–145\;{Å}^{2}\;molecule^{-1}$, with systematically larger areas observed at pH 5.5 than at pH 8.0.

Consequently, interfacial accommodation of Vipoxin or its isolated subunits cannot be described in terms of a single fixed lipid area. Instead, the geometric mismatch between the protein footprint and the pressure-dependent SAPC area implies that protein adsorption or partial insertion requires local lipid rearrangement, displacement, or redistribution within an expanded and laterally heterogeneous monolayer. These estimates should therefore be regarded as geometric approximations. The effective interfacial footprint depends on molecular orientation, depth of insertion, conformational flexibility, as well as contributions from hydration shells and associated lipid molecules. Nevertheless, the pronounced disparity between the cross-sectional dimensions of Vipoxin (or its subunits) and SAPC provides a physically plausible explanation for the negative ΔA(t) signals observed at low surface pressures. Under expanded conditions, the monolayer possesses sufficient free area to accommodate adsorption and partial insertion events, resulting in measurable barrier expansion under barostatic control. Conversely, at elevated surface pressures, where lateral packing constraints are stronger, deep insertion becomes energetically less favorable, and the ΔA(t) signal increasingly reflects hydrolysis-coupled lipid removal rather than adsorption-driven expansion.

Collectively, these results demonstrate that the surface pressure governs the balance between adsorption and productive hydrolysis. Expanded monolayers could favor incorporation of large protein molecules and barostatic area expansion, whereas compressed films promote measurable lipolysis reflected by positive ΔA(t) responses [32]. Moreover, this pressure dependence must be interpreted in the context of the specific molecular architecture of the SAPC substrate. In contrast to saturated phospholipids that readily form tightly packed condensed phases, SAPC contains a polyunsaturated arachidonoyl chain (20:4) at the sn-2 position. The presence of four cis double bonds introduces pronounced conformational disorder and prevents efficient crystalline packing. Structural studies have shown that arachidonoyl chains can adopt kinked or hairpin-like conformations with a large effective cross-sectional area, thereby generating increased free area and interfacial voids within lipid assemblies [33]. Consequently, compression of SAPC monolayers does not produce a uniformly rigid interface but instead promotes a structurally heterogeneous state characterized by enhanced lateral compressibility and a higher density of packing defects. Such defects are known to play a central role in interfacial enzymology by facilitating enzyme penetration, interfacial activation, and productive substrate binding. Within this framework, the pressure-dependent enhancement of catalytic response observed at higher surface pressures is consistent with defect-mediated accessibility of the sn-2 ester bond. This interpretation is compatible with the “slotting” mechanism proposed for phospholipase A_2_ catalysis, in which substrate recognition is governed by geometric constraints requiring a sharply kinked sn-2 chain to properly align the ester carbonyl within the catalytic site, while sterically excluding the sn-1 chain. Although the present experiments do not directly resolve molecular conformations, the known conformational flexibility of polyunsaturated arachidonoyl chains provides a plausible structural basis for the observed pressure sensitivity [34].

In this context, VBC exhibits the highest interfacial activity and is influenced mainly by monolayer packing, with only limited sensitivity to acidic versus basic subphase conditions. Vipoxin, however, displays a markedly stronger pH dependence, consistent with VAC-mediated modulation of enzyme-interface coupling. VAC subunit alone behaves predominantly as an adsorption-active component whose interfacial influence becomes mechanically detectable primarily at elevated lateral pressures, where SAPC chain reorganization and defect redistribution are most pronounced. Importantly, qualitative comparisons based solely on the visual steepness of ΔA(t) traces may be misleading across different phospholipid packing regimes. Monolayer compression affects not only enzyme accessibility to the lipid substrate but also the magnitude of the macroscopic ΔA(t) response recorded under barostatic conditions. For this reason, enzyme activity is evaluated quantitatively using the global kinetic parameter $Q_{m}$ (Section 3.4), which provides an operational descriptor of interfacial quality that is less sensitive to packing-dependent area effects.

### 3.3. Effect of Subphase pH on Interfacial Activity

**Figure 5:**
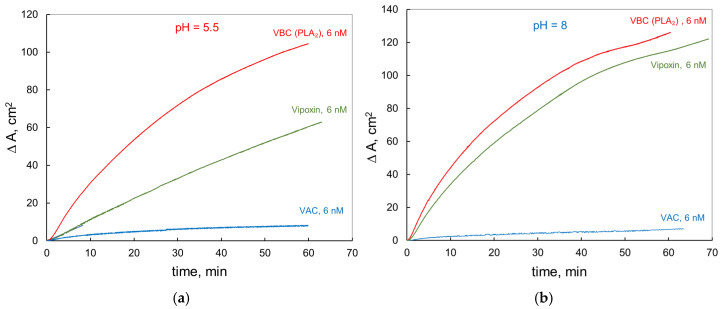
Effect of subphase pH on the interfacial activity of Vipoxin and its individual subunits at constant surface pressure. Time dependence of the compensated monolayer area change, ΔA(t), during interaction of Vipoxin protein forms with SAPC monolayers recorded under strictly barostatic conditions at $\pi =20\;mN\;m^{-1}$ following injection into the subphase t = 0: (**a**) acidic subphase (acetate buffer, pH 5.5); (**b**) basic subphase (Tris-HCl buffer, pH 8.0). Measurements were performed at a fixed concentration of 6 nM for heterodimeric Vipoxin, VBC (PLA_2_), and VAC. Under constant surface pressure, positive ΔA(t) responses indicate net removal of surface-active material from the interface and are interpreted within the interfacial kinetic framework rather than as direct product-specific measurements.

To decouple chemical effects from variations in monolayer packing, enzymatic hydrolysis experiments were performed at a fixed surface pressure ($\pi \;=\;20\;mN\;m^{-1}$). Under these strictly barostatic conditions, the SAPC monolayer was maintained at constant lateral pressure while the pH of the subphase was varied between acidic (pH 5.5) and basic (pH 8.0) environments. Figure 5 presents representative time-dependent profiles of the compensated monolayer area change, ΔA(t), recorded immediately following injection of Vipoxin, VBC, or VAC into the subphase (t = 0). To examine how subphase conditions influence the interfacial response independently of changes in applied surface pressure, experiments were performed at a fixed surface pressure of $\pi \;=\;20\;mN\;m^{-1}$. Under these strictly barostatic conditions, the SAPC monolayer was maintained at constant lateral pressure while the subphase was varied between an acidic acetate buffer (pH 5.5) and a basic Tris-HCl buffer (pH 8.0). This design allows comparison of the interfacial behavior of Vipoxin protein forms under defined acidic and basic conditions, although the observed differences cannot be attributed exclusively to pH because the buffer species also differ. Figure 5 presents representative time-dependent profiles of the compensated monolayer area change, ΔA(t), recorded immediately following injection of Vipoxin, VBC, or VAC into the subphase (t = 0)).

As outlined in Section 3.1, under constant surface pressure the ΔA(t) signal represents the net barostatic response of the interface. Positive values indicate dominant lipid removal processes, whereas negative values reflect adsorption-driven area expansion. Accordingly, ΔA(t) does not directly measure catalytic turnover, but the overall mechanical compensation required to maintain constant π. The experimental data demonstrate that the interfacial response of Vipoxin is strongly modulated by subphase pH. At acidic pH (5.5; Figure 5a), the isolated PLA_2_ (VBC) exhibits a steep positive ΔA(t) response, characterized by rapid initial increase followed by progressive deceleration, indicative of nonlinear interfacial kinetics.

In comparison, the heterodimeric Vipoxin complex exhibits a positive but substantially reduced ΔA(t) response relative to the isolated catalytic subunit. Although the temporal profile appears more nearly linear over the experimental time window, the reduced magnitude indicates that association with VAC significantly alters the effective interfacial behavior of the catalytic subunit under acidic conditions. The persistence of a positive ΔA(t) signal remains consistent with ongoing productive interfacial turnover, albeit at lower apparent efficiency. VAC is structurally classified as catalytically inactive due to the substitution of the conserved active-site His48 residue by Gln48, a mutation predicted to abolish the canonical general-base mechanism of sPLA_2_ [12].

Within this classical framework, the absence of His48 precludes efficient nucleophilic water activation and therefore lipid ester bond cleavage. Nevertheless, the detection of a small but reproducible positive ΔA(t) signal at pH 5.5 and high surface pressure raises the question of whether VAC is strictly inert under all interfacial conditions. While ΔA(t) measurements do not provide direct molecular evidence of hydrolysis, the observed response indicates that VAC participates in interfacial processes capable of producing net material redistribution at the lipid interface.

One possible explanation is that acidic pH alters the physicochemical environment of the interface, modifying protein adsorption, hydration, or lipid organization in ways that enhance VAC-mediated interfacial perturbation. However, because ΔA(t) measurements do not directly detect reaction products, the present data cannot distinguish between trace catalytic turnover and non-catalytic lipid displacement or interfacial restructuring [35].

Alternatively, and more conservatively, VAC-induced ΔA(t) response may arise from adsorption-driven lipid displacement, defect stabilization, or protein-mediated interfacial reorganization without bond cleavage. Distinguishing between these possibilities requires orthogonal product-specific assays [36].

Regardless of the underlying mechanism, the data demonstrate that VAC exhibits measurable interfacial functionality that depends on the subphase conditions and is enhanced under acidic acetate conditions. This observation is consistent with the emerging concept that pseudoenzymes may retain latent or context-dependent activities and supports the interpretation of VAC as an environmentally responsive modulatory subunit rather than a purely inert structural partner [37,38].

Taken together, these observations indicate a clear subphase-condition-dependent modulation of the functional contributions within the Vipoxin complex at lipid interfaces. The catalytic VBC subunit retains high interfacial activity under both acidic acetate and basic Tris-HCl conditions, consistent with preservation of its hydrolytic competence across the two tested subphase environments.

In contrast, the VAC subunit exhibits a distinctly pH-sensitive interfacial response. Its measurable ΔA(t) contribution, selectively enhanced at pH 5.5, indicates that VAC participates in interfacial processes that become more prominent under acidic conditions. Although VAC is classically regarded as catalytically inactive, the observed behavior is consistent with a conditionally expressed modulatory role influencing interfacial organization, lipid packing, and enzyme-membrane coupling.

While the present barostatic measurements do not provide molecular-level resolution of the underlying mechanism, the data do not exclude the possibility that the combination of acidic acetate conditions and high lateral pressure alters the interfacial behavior of VAC in a way that produces a weak positive ΔA(t) response. This response may reflect adsorption-driven lipid displacement, interfacial restructuring, or, less likely, extremely low-efficiency catalytic-like events that cannot be verified without product-specific assays. Regardless of its precise origin, VAC clearly contributes to the interfacial dynamics of the Vipoxin system in a subphase-condition-dependent manner.

These findings support the interpretation of VAC as a pseudoenzymatic regulatory component whose functional influence emerges at the membrane interface primarily through modulation of interfacial interactions rather than through dominant catalytic turnover.

### 3.4. Comparative Interfacial Efficiency Quantified by the Q_m_ Parameter

Although the experimental ΔA(t) data reflect the macroscopic mechanical compensation of the monolayer under barostatic conditions, quantitative comparison across different surface pressures and subphase pH values requires a normalized interfacial kinetic descriptor. Accordingly, the experimentally derived interfacial quality parameter $Q_{m}$(exp), as defined in Section 2.4, was used as the principal comparative measure of enzyme behavior at the lipid interface.

Table 2 summarizes $Q_{m}$ values obtained using two complementary analytical approaches: (i) the experimentally derived parameter $Q_{m}$(exp), obtained directly from the initial slopes of the ΔA(t) traces, and (ii) the formal model-derived parameter $Q_{m}$(formal), calculated using the Verger–Panaiotov interfacial kinetic framework. While the formal treatment explicitly incorporates catalytic turnover together with adsorption–desorption equilibria, interfacial activation, penetration dynamics, and product removal processes, its application to SAPC hydrolysis is complicated by uncertainties associated with estimating the effective surface concentration and molecular areas of accumulating long-chain hydrolysis products. These limitations may introduce systematic deviations into model-derived constants. Because $Q_{m}$ is derived directly from measured barostatic responses without requiring such assumptions, it was prioritized as the principal descriptor for comparative analysis. The resulting comparison reveals a clear subphase-condition-dependent divergence between the heterodimeric Vipoxin complex and its isolated subunits. At pH 8.0, Vipoxin and the catalytic subunit VBC display comparable interfacial quality across the investigated surface pressure range, indicating that under basic, near-physiological conditions, association with VAC does not measurably diminish the effective interfacial activity of VBC. In contrast, at pH 5.5, the isolated VBC subunit retains high interfacial quality, whereas Vipoxin exhibits substantially reduced $Q_{m}$(exp) values. This divergence suggests that acidic pH alters the functional coupling between VAC and VBC, leading to attenuation of the interfacial quality of the heterodimer. Notably, VAC itself exhibits weak but reproducible $Q_{m}$(exp) values at the highest surface pressure. Although VAC is classically regarded as catalytically inactive, the emergence of a measurable interfacial response—particularly under acidic conditions—indicates its participation in pressure-dependent interfacial processes.

**Figure 6:**
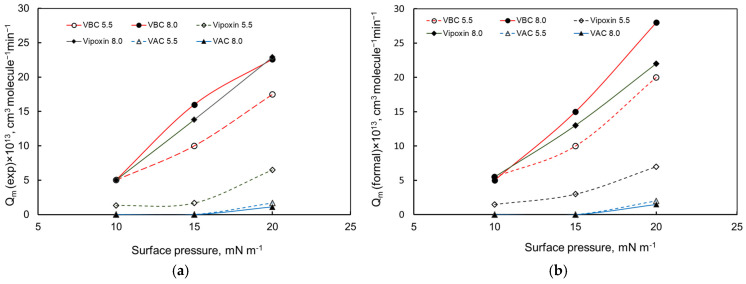
Pressure dependence of the interfacial quality parameter ($Q_{m}$) for Vipoxin and its isolated subunits. (**a**) $Q_{m}(\exp)$ values derived directly from the initial slopes of the experimental ($\Delta A\left(t\right))$ traces recorded during barostatic interfacial kinetic experiments with SAPC monolayers. (**b**) $Q_{m}(\mathrm{formal})$ values obtained from analysis based on the Verger–Panaiotov interfacial kinetic formalism. Data are shown for the heterodimeric Vipoxin complex, the catalytic subunit VBC, and the catalytically impaired subunit VAC under basic Tris-HCl conditions (pH 8.0) and acidic acetate conditions (pH 5.5) across three initial surface pressures: $10,\;15,\;and\;20\;mN\;m^{-1}$. Data represent mean values from at least three independent measurements. Because the experimental variability was within approximately 1% of the mean values, error bars were omitted for clarity.

Figure 6 provides a graphical representation of $Q_{m}$ as a function of surface pressure for Vipoxin and its isolated subunits at both pH values, facilitating visualization of pressure-dependent trends and highlighting distinct regimes governing enzyme function at the interface.

At pH 8.0, VBC exhibits a concave, saturation-type $Q_{m}(\pi )$ profile (Figure 6a). This behavior is characteristic of penetration- or accommodation-limited kinetics, where increasing lateral pressure progressively restricts enzyme insertion and optimal alignment. In contrast, Vipoxin displays an approximately linear $Q_{m}(\pi )$ dependence, indicating that association with VAC modifies the pressure response of the catalytic subunit and mitigates interfacial constraints. This trend is consistent with VAC acting as an interfacial stabilizer rather than an inhibitor of catalytic activity [7].

At pH 5.5, both Vipoxin and VBC exhibit convex $Q_{m}(\pi )$ profiles. Convexity suggests pressure-enhanced interfacial quality, commonly associated with interfacial activation or defect-mediated catalysis. Compression likely generates transient packing defects or domain boundaries that facilitate productive enzyme–substrate interactions. The shift from concave to convex behavior therefore reflects a transition from penetration-limited to defect-assisted interfacial kinetics. Figure 6b reproduces the same qualitative trends, supporting the conclusion that the pressure- and subphase-condition-dependent variations are reproducible features of the VBC–SAPC monolayer system under the tested experimental conditions. Structural insights further support this interpretation. Crystallographic analysis of Vipoxin [12] revealed strong homology between VAC and the catalytic PLA_2_ subunit, despite the critical His48 → Gln48 substitution associated with catalytic inactivation. Both subunits retain a conserved hydrophobic channel, indicating preserved lipid interaction potential. Additionally, the Asp49 residue of VBC forms a stabilizing salt bridge with Lys69 of VAC, suggesting VAC contributes to electrostatic stabilization of regions implicated in interfacial activation and Ca^2+^-dependent catalysis.

Collectively, $Q_{m}$ analysis demonstrates that VAC contributes to interfacial dynamics in a manner dependent on pH and lateral pressure. While its catalytic competence remains negligible relative to VBC, its measurable influence on interfacial quality supports classification within the family of pseudoenzymes. The pressure-dependent linearization observed for Vipoxin indicates that VAC functions primarily as an interfacial regulatory subunit that stabilizes enzyme-membrane coupling and modulates catalytic efficiency under varying physicochemical conditions.

$Q_{m}$ captures the integrated influence of catalytic turnover where applicable, interfacial penetration, adsorption–desorption equilibria, and adaptation of the Vipoxin protein forms to lipid packing. The distinct pressure- and subphase-condition-dependent trends observed for Vipoxin, VBC, and VAC indicate that VAC modulates interfacial quality predominantly through interfacial accommodation and regulatory coupling rather than through dominant catalysis.

## 4. Discussion

Previous studies have shown that Vipoxin exhibits a coordinated functional response across enzymatic and non-catalytic activities, reflecting interplay between its catalytic (VBC) and non-catalytic (VAC) subunits during multistep lipid processing [27,39]. In the present work, we extend this analysis to arachidonic-acid-containing phospholipids using SAPC monolayers as a controlled interfacial model, with the aim of elucidating how substrate composition and physicochemical conditions modulate Vipoxin function.

The present results demonstrate a strong dependence of Vipoxin activity on the structural properties of the lipid interface. Owing to the polyunsaturated arachidonoyl chain, SAPC monolayers remain disordered and heterogeneous even under compression, retaining a high density of transient packing defects. These defects are known to facilitate enzyme penetration and interfacial activation, suggesting that the strong pressure dependence observed here reflects a defect-mediated mechanism in which local lipid organization, rather than surface pressure alone, governs access to the sn-2 ester bond. This interpretation is consistent with structural models such as the “slotting” mechanism of phospholipase A_2_, although the present data do not directly resolve molecular conformations.

Within this structurally dynamic interfacial environment, the role of the VAC subunit emerges as highly context-dependent. While VAC is classically regarded as catalytically impaired due to the His48 → Gln48 substitution, the present results show that it induces reproducible interfacial effects under specific subphase conditions. Its contribution becomes most pronounced under acidic acetate conditions and elevated lateral pressure, where the lipid interface is both highly constrained and defect-rich. Because barostatic $\Delta A\left(t\right)$ measurements reflect an integrated macroscopic response, these area changes are most conservatively interpreted as adsorption-driven lipid displacement, defect stabilization, or protein-mediated interfacial restructuring. Although extremely low-efficiency or non-canonical catalytic-like events cannot be definitively ruled out, they cannot be verified without product-specific assays. The data therefore primarily support a mechanism in which VAC physically interacts with the monolayer and perturbs local lipid packing without requiring conventional enzymatic bond cleavage. The conserved hydrophobic channel of VAC may contribute to this behavior by accommodating bulky arachidonoyl chains, but this possibility remains a mechanistic interpretation rather than direct structural evidence.

To assess whether the magnitude of the observed barostatic response can be rationalized solely in terms of geometric occupation of the interface, an approximate estimate of the effective interfacial footprint of the VAC subunit was considered. Based on crystallographic dimensions, a single VAC molecule is expected to occupy on the order of several hundred ${Å}^{2}$ at the interface, depending on its orientation and degree of insertion. Using the experimentally determined final values of ΔA(t), an apparent number of interfacially associated VAC molecules can be estimated according to $N_{app}=|\Delta A|/a_{eff}$, where $a_{eff}$ represents an effective footprint. Comparison with the total number of VAC molecules available in the subphase (determined from bulk concentration and compartment volume) shows that the resulting values approach or, in some cases, exceed the total number of molecules present. This discrepancy indicates that the negative ΔA(t) response cannot be interpreted solely in terms of direct molecular occupation of the interface. Rather, ΔA(t) reflects a combined contribution of protein adsorption, partial insertion, and lipid reorganization within the SAPC monolayer. The calculated $N_{app}$ should therefore be regarded as an upper-bound estimate of interfacial occupancy. These observations are consistent with protein-mediated lipid reorganization and defect stabilization, supporting a predominantly non-catalytic role for VAC at the membrane interface.

Taken together, these findings are consistent with the interpretation of VAC as a pseudoenzymatic regulatory component whose functional influence emerges at the membrane interface. Rather than acting as a simple inhibitor, VAC may function as an interfacial modulatory component. The partial linearization of the $Q_{m}\left(\pi \right)$ profiles for the heterodimer under basic Tris-HCl conditions is consistent with reduced penetration-limited saturation compared with the isolated VBC subunit at high surface pressures. In this way, VAC appears to alter how the heterodimer accommodates the SAPC interface, modulating the complex response to lateral packing and subphase conditions. This view aligns with the broader concept of pseudoenzymes as catalytically impaired homologs that retain important context-dependent functions, particularly in systems governed by multiparametric interfacial processes rather than by a single catalytic step.

The application of the experimentally derived kinetic parameter $Q_{m}\left(\exp\right)$ provides a comparative descriptor that further supports this framework. Rather than serving as absolute mechanistic proof, the $Q_{m}$ profiles support the view that Vipoxin behaves as a coupled interfacial system in which VAC modulates the effective interfacial activity of VBC as a function of surface pressure and subphase conditions. Thus, VAC appears to influence the interaction of the heterodimer with the lipid interface and thereby contributes to the overall functional response of the complex. This highlights the importance of treating lipid–protein interactions as emergent properties of the interface, where enzymatic activity, membrane structure, and the local physicochemical environment are intimately coupled. In this context, Vipoxin represents a useful model for understanding how catalytically impaired protein homologs acquire biological significance through modulation of membrane-associated processes.

Finally, several methodological limitations of the present study should be acknowledged. The barostatic ΔA(t) signal represents an integrated interfacial response and does not constitute direct chemical proof of catalytic turnover. Product-resolved assays, such as LC-MS analysis of released arachidonic acid, pH-stat measurements, fluorescent lipid reporters, or inhibitor-based validation, were not performed in parallel with the barostatic monolayer experiments. Consequently, the ΔA(t) responses, particularly those observed for VAC, cannot unambiguously distinguish between phospholipid hydrolysis, lipid reorganization, adsorption-driven displacement, or trace catalytic-like events. Instead, these responses are interpreted as integrated interfacial signals reflecting the combined contributions of adsorption, penetration, lipid displacement, catalytic turnover where applicable, product accumulation or removal, and monolayer reorganization. Future investigations combining barostatic monolayer measurements with product-specific assays, inhibition of the catalytic VBC subunit, and advanced fluorescence-based interfacial techniques, including total internal reflection fluorescence (TIRF), Förster resonance energy transfer (FRET), and fluorescently labeled lipid substrates, will be required to resolve the relative contributions of hydrolysis and non-catalytic interfacial restructuring and to provide direct molecular insight into Vipoxin subunit interactions, membrane binding dynamics, and pseudoenzymatic regulation at lipid interfaces.

## 5. Conclusions

This study demonstrates that the functional behavior of Vipoxin at arachidonic-acid-containing membrane interfaces is governed not only by the intrinsic catalytic capability of its PLA_2_ subunit but also by the physicochemical context imposed by the lipid substrate. SAPC monolayers, enriched in packing disorder and transient defects due to the flexible arachidonoyl chain, create a dynamic interfacial environment in which surface pressure, pH, and local heterogeneity collectively regulate enzyme accessibility and activity. Within this environment, the VAC subunit—traditionally considered catalytically inactive—exhibits distinct pH- and pressure-dependent interfacial effects. Together with the linearized Q_m_ response of the heterodimer, these findings demonstrate that VAC acts as a pseudoenzymatic regulatory component that modulates VBC penetration, interfacial activation, and effective catalytic performance. Vipoxin therefore behaves as a cooperative two-subunit system whose emergent functional properties arise from interfacial coupling rather than simple inhibition, providing a mechanistic framework for how pseudoenzymes regulate phospholipase activity at biologically relevant membrane interfaces.

## Figures and Tables

**Table 1:** Interfacial mechanical properties of phospholipid monolayers evaluated in the bilayer-equivalent surface pressure regime (π≈20–30 mN m^−1^).

Monolayer	Subphase pH	Area at $25\;mN\;m^{-1}$ (${Å}^{2}\;Molecule^{-1}$)	$C_{s}^{-1}$ ($mN\;m^{-1}$)	Interfacial State
DPPC	—	~52.5	~105	Condensed/ordered
SAPC	8.0	~80.0	~80	Liquid-expanded (LE)
SAPC	5.5	~100.0	~100	Expanded LE

**Table 2:** Interfacial quality parameter $Q_{m}$ of Vipoxin and its isolated subunits during SAPC monolayer hydrolysis.

Vipoxin and/or Subunits	π	pH 5.5		pH 8.0	
		${Q_{m}\left(exp\right)}^{*}\times 10^{13}$	${Q_{m}\left(Formal\right)}^{**}\;{\times 10}^{13}$	$Q_{m}(exp)\times 10^{13}$	$Q_{m}(Formal)\times 10^{13}$
	($mN\;m^{-1}$)	$\left({cm}^{3}\;{molecules}^{-1}\;{min}^{-1}\right)$			
VBC	10	4.59	5.50	4.50	5.00
	15	10.00	10.00	14.50	15.00
	20	17.50	20.00	25.00	28.00
Vipoxin	10	1.32	1.50	5.00	5.50
	15	2.68	3.00	12.50	13.00
	20	6.45	7.00	20.00	22.00
VAC	10	nd	nd	nd	nd
	15	nd	nd	nd	nd
	20	1.67	2.00	1.30	1.50

* $Q_{m}(\exp)$ derived directly from initial slopes $\Delta A\left(t\right)$. ** $Q_{m}(\mathrm{formal})$ obtained using Verger–Panaiotov model. “nd” = not detectable or negligible activity.

## Data Availability

The original contributions presented in this study are included in the article. Further inquiries can be directed to the corresponding author.

## Abbreviations

The following abbreviations are used in this manuscript:

sPLA_2_	Secreted phospholipase A_2_
VBC	Catalytically active Vipoxin phospholipase A_2_ subunit
VAC	Catalytically inactive acidic Vipoxin subunit
SAPC	1-Stearoyl-2-arachidonoyl-sn-glycero-3-phosphocholine
DPPC	1,2-Dipalmitoyl-sn-glycero-3-phosphocholine
BMP	Bis(monoacylglycero)phosphate
LE	Liquid-expanded phase
LC	Liquid-condensed phase
LE–LC	Liquid-expanded/liquid-condensed phase coexistence
β-CD	β-Cyclodextrin
$Q_{m}$	Interfacial quality parameter
ΔA(t)	Time-dependent compensated monolayer area change
ES	Enzyme–substrate complex
TIRF	Total internal reflection fluorescence
FRET	Förster resonance energy transfer

## References

[1] Castro-Amorim J., Novo De Oliveira A., Da Silva S.L., Soares A.M., Mukherjee A.K., Ramos M.J., Fernandes P.A. (2023). Catalytically Active Snake Venom PLA_2_ Enzymes: An Overview of Its Elusive Mechanisms of Reaction: Miniperspective. J. Med. Chem..

[2] Kini R.M. (2003). Excitement Ahead: Structure, Function and Mechanism of Snake Venom Phospholipase A_2_ Enzymes. Toxicon.

[3] Cho W., Markowitz M.A., Kezdy F.J. (1988). A New Class of Phospholipase A_2_ Substrates: Kinetics of the Phospholipase A_2_ Catalyzed Hydrolysis of 3-(Acyloxy)-4-Nitrobenzoic Acids. J. Am. Chem. Soc..

[4] Van Eijk J.H., Verheij H.M., Dijkman R., De Haas G.H. (1983). Interaction of Phospolipase A_2_ Form *Naja Melanoleuca* Sanke Venom with Meoomeric Substrate Analogs: Activation of Enzyme by Protein-protein or Liquid-Protein Interactions?. Eur. J. Biochem..

[5] Oliveira A.L., Viegas M.F., Da Silva S.L., Soares A.M., Ramos M.J., Fernandes P.A. (2022). The Chemistry of Snake Venom and Its Medicinal Potential. Nat. Rev. Chem..

[6] Aleksiev B., Tchorbanov B. (1976). Action on Phosphatidylcholine of the Toxic Phospholipase A_2_ from the Venom of Bulgarian Viper (*Vipera Ammodytes Ammodytes*). Toxicon.

[7] Tchorbanov B., Grishin E., Aleksiev B., Ovchinnikov Y. (1978). A Neurotoxic Complex from the Venom of the Bulgarian Viper (*Vipera Ammodytes Ammodytes*) and a Partial Amino Acid Sequence of the Toxic Phospholipase A_2_. Toxicon.

[8] Georgieva D., Risch M., Kardas A., Buck F., Von Bergen M., Betzel C. (2008). Comparative Analysis of the Venom Proteomes of *Vipera Ammodytes Ammodytes* and *Vipera Ammodytes Meridionalis*. J. Proteome Res..

[9] Petrova S.D., Atanasov V.N., Balashev K. (2012). Vipoxin and Its Components: Structure-Function Relationship. Adv. Protein Chem. Struct. Biol..

[10] Mancheva I., Kleinschmidt T., Aleksiev B., Braunitzer G. (1984). Sequence Homology between Phospholipase and Its Inhibitor in Snake Venom. The Primary Structure of the Inhibitor of Vipoxin from the Venom of the Bulgarian Viper (*Vipera Ammodytes Ammodytes*, Serpentes). Hoppe-Seyler’s Z. Für Physiol. Chem..

[11] Mancheva I., Kleinschmidt T., Aleksiev B., Braunitzer G. (1987). Sequence Homology between Phospholipase and Its Inhibitor in Snake Venom. The Primary Structure of Phospholipase A_2_ of Vipoxin from the Venom of the Bulgarian Viper (*Vipera Ammodytes Ammodytes*, Serpentes). Biol. Chem. Hoppe-Seyler.

[12] Banumathi S., Rajashankar K.R., Nötzel C., Aleksiev B., Singh T.P., Genov N., Betzel C. (2001). Structure of the Neurotoxic Complex Vipoxin at 1.4 Å Resolution. Acta Crystallogr. Sect. D Biol. Crystallogr..

[13] Perbandt M., Wilson J.C., Eschenburg S., Mancheva I., Aleksiev B., Genov N., Willingmann P., Weber W., Singh T.P., Betzel C. (1997). Crystal Structure of Vipoxin at 2.0 Å: An Example of Regulation of a Toxic Function Generated by Molecular Evolution. FEBS Lett..

[14] Aleksiev B., Tchorbanov B. (1981). A Simple Procedure for the Isolation of Vipoxin P a Neurotoxin with Phospholipase A_2_ Activity from the Venom of the Bulgarian Viper (*Vipera ammodytes*). J. Appl. Biochem..

[15] Devedjiev Y., Popov A., Atanasov B., Bartunik H.-D. (1997). X-Ray Structure at 1.76 Å Resolution of a Polypeptide Phospholipase A_2_ Inhibitor. J. Mol. Biol..

[16] Atanasov V., Petrova S., Mitewa M. (2009). HPLC Assay of Phospholipase A_2_ Activity Using Low-Temperature Derivatization of Fatty Acids. Anal. Lett..

[17] Atanasov V.N., Stoykova S., Goranova Y., Mitewa M., Petrova S. (2012). Acute Toxicity of Vipoxin and Its Components: Is the Acidic Component an “Inhibitor” of PLA_2_ Toxicity?. Interdiscip. Toxicol..

[18] Rouault M., Rash L.D., Escoubas P., Boilard E., Bollinger J., Lomonte B., Maurin T., Guillaume C., Canaan S., Deregnaucourt C. (2006). Neurotoxicity and Other Pharmacological Activities of the Snake Venom Phospholipase A_2_ OS_2_: The N-Terminal Region Is More Important Than Enzymatic Activity. Biochemistry.

[19] Faure G., Harvey A.L., Thomson E., Saliou B., Radvanyi F., Bon C. (1993). Comparison of Crotoxin Isoforms Reveals That Stability of the Complex Plays a Major Role in Its Pharmacological Action. Eur. J. Biochem..

[20] Faure G., Gowda V.T., Maroun R.C. (2007). Characterization of a Human Coagulation Factor Xa-Binding Site on Viperidae Snake Venom Phospholipases A_2_ by Affinity Binding Studies and Molecular Bioinformatics. BMC Struct. Biol..

[21] Mora-Obando D., Fernández J., Montecucco C., Gutiérrez J.M., Lomonte B. (2014). Synergism between Basic Asp49 and Lys49 Phospholipase A_2_ Myotoxins of Viperid Snake Venom In Vitro and In Vivo. PLoS ONE.

[22] Zambelli V.O., Chioato L., Gutierrez V.P., Ward R.J., Cury Y. (2017). Structural Determinants of the Hyperalgesic Activity of Myotoxic Lys49-Phospholipase A_2_. J. Venom. Anim. Toxins Incl. Trop. Dis..

[23] Doltchinkova V., Stoylov S., Angelova P.R. (2021). Viper Toxins Affect Membrane Characteristics of Human Erythrocytes. Biophys. Chem..

[24] Jenko-Pražnikar Z., Petan T., Pungerčar J. (2013). Ammodytoxins Efficiently Release Arachidonic Acid and Induce Apoptosis in a Motoneuronal Cell Line in an Enzymatic Activity-Dependent Manner. NeuroToxicology.

[25] Verger R., Mieras M.C.E., De Haas G.H. (1973). Action of Phospholipase A at Interfaces. J. Biol. Chem..

[26] Mircheva K., Minkov I., Ivanova T., Panaiotov I., Proust J.E., Verger R. (2008). Comparative Study of Lipolysis by PLA_2_ of DOPC Substrates Organized as Monolayers, Bilayer Vesicles and Nanocapsules. Colloids Surf. B Biointerfaces.

[27] Mircheva K., Petrova S.D., Ivanova T., Panaiotov I., Balashev K.T. (2019). Action of Vipoxin and Its Separated Components on Monomolecular Film of Dilauroylphosphatidylcholine at the Air/Water Interface. Colloids Surf. A Physicochem. Eng. Asp..

[28] Mohwald H. (1990). Phospholipid and Phospholipid-Protein Monolayers at the Air/Water Interface. Annu. Rev. Phys. Chem..

[29] Duncan S.L., Larson R.G. (2008). Comparing Experimental and Simulated Pressure-Area Isotherms for DPPC. Biophys. J..

[30] Heffern C.T.R., Pocivavsek L., Birukova A.A., Moldobaeva N., Bochkov V.N., Lee K.Y.C., Birukov K.G. (2013). Thermodynamic and Kinetic Investigations of the Release of Oxidized Phospholipids from Lipid Membranes and Its Effect on Vascular Integrity. Chem. Phys. Lipids.

[31] Tsubone T.M., de Oliveira Junior P.N., Scanavachi G., dos Santos V.F., da Cunha A.R., Ramos A.P., Soares T.A., Itri R. (2026). Acidic pH Modulates Headgroup Orientation and Packing in Bis(Monoacylglycero)Phosphate Bilayers. ACS Phys. Chem. Au.

[32] Grainger D.W., Reichert A., Ringsdorf H., Salesse C. (1990). Hydrolytic Action of Phospholipase A_2_ in Monolayers in the Phase Transition Region: Direct Observation of Enzyme Domain Formation Using Fluorescence Microscopy. BBA—Biomembr..

[33] Applegate K.R., Glomset J.A. (1988). Computer-Based Modeling of the Conformation and Packing Properties of Docosahexaenoic Acid. J. Lipid Res..

[34] White S.P., Scott D.L., Otwinowski Z., Gelb M.H., Sigler P.B. (1990). Crystal Structure of Cobra-Venom Phospholipase A_2_ in a Complex with a Transition-State Analogue. Science.

[35] Dall’Acqua W., Carter P. (2000). Substrate-assisted Catalysis: Molecular Basis and Biological Significance. Protein Sci..

[36] Swairjo M.A., Roberts M.F., Campos M.-B., Dedman J.R., Seaton B.A. (1994). Annexin V Binding to the Outer Leaflet of Small Unilamellar Vesicles Leads to Altered Inner Leaflet Properties: 31P- and 1H-NMR Studies. Biochemistry.

[37] Murphy J.M., Czabotar P.E., Hildebrand J.M., Lucet I.S., Zhang J.-G., Alvarez-Diaz S., Lewis R., Lalaoui N., Metcalf D., Webb A.I. (2013). The Pseudokinase MLKL Mediates Necroptosis via a Molecular Switch Mechanism. Immunity.

[38] Ribeiro A.J.M., Holliday G.L., Furnham N., Tyzack J.D., Ferris K., Thornton J.M. (2018). Mechanism and Catalytic Site Atlas (M-CSA): A Database of Enzyme Reaction Mechanisms and Active Sites. Nucleic Acids Res..

[39] Mircheva K., Grozev N., Petrova S.D., Ivanova T., Panaiotov I., Balashev K.T. (2021). The Enzymatic Action of Vipoxin on Insoluble Long-Chain Phospholipid Langmuir Monolayers. Colloids Surf. A Physicochem. Eng. Asp..

